# The Role of N-Methyl-D-Aspartate Receptor Neurotransmission and Precision Medicine in Behavioral and Psychological Symptoms of Dementia

**DOI:** 10.3389/fphar.2019.00540

**Published:** 2019-05-22

**Authors:** Chieh-Hsin Lin, Hsien-Yuan Lane

**Affiliations:** ^1^ Department of Psychiatry, Kaohsiung Chang Gung Memorial Hospital, Chang Gung University College of Medicine, Kaohsiung, Taiwan; ^2^ School of Medicine, Chang Gung University, Taoyuan, Taiwan; ^3^ Graduate Institute of Biomedical Sciences, China Medical University, Taichung, Taiwan; ^4^ Department of Psychiatry and Brain Disease Research Center, China Medical University Hospital, Taichung, Taiwan; ^5^ Department of Psychology, College of Medical and Health Sciences, Asia University, Taichung, Taiwan

**Keywords:** behavioral and psychological symptoms of dementia, Alzheimer’s disease, N-methyl-D-aspartate receptor, precision medicine, gender difference

## Abstract

While the world’s population is aging, the prevalence of dementia and the associated behavioral and psychological symptoms of dementia (BPSD) rises rapidly. BPSD are associated with worsening of cognitive function and poorer prognosis. No pharmacological treatment has been approved to be beneficial for BPSD to date. Dysfunction of the N-methyl-D-aspartate receptor (NMDAR)-related neurotransmission leads to cognitive impairment and behavioral changes, both of which are core symptoms of BPSD. Memantine, an NMDAR partial antagonist, is used to treat moderate to severe Alzheimer’s disease (AD). On the other hand, a D-amino acid oxidase inhibitor improved early-phase AD. Whether to enhance or to attenuate the NMDAR may depend on the phases of dementia. It will be valuable to develop biomarkers indicating the activity of NMDAR, particularly in BPSD. In addition, recent reports suggest that gender difference exists in the treatment of dementia. Selecting subpopulations of patients with BPSD who are prone to improvement with treatment would be important. We reviewed literatures regarding the treatment of BPSD, focusing on the NMDAR-related modulation and precision medicine. Future studies examining the NMDAR modulators with the aid of potential biomarkers to tailor the treatment for individualized patients with BPSD are warranted.

## Introduction

Dementia is a severe neurodegenerative disorder, affecting 1.5% of the population at the age of 65, and >20% at the age of 85 (Ritchie and Kildea, 1995). The morbidity and mortality of dementia are high. For elderly people aged between 65 and 85 years, the prevalence rate of dementia doubles every 5 years (Fratiglioni et al., 1999). About 24.3 million people worldwide were diagnosed with dementia in 2005 (Ferri et al., 2005). In 2010, 35 million people had dementia worldwide (Brodaty et al., 2011). The population of people with dementia is estimated to be 65 million by 2030 and 113 million by 2050 (Brodaty et al., 2011). The prevalence rate has been rising fast in the rapid-aging society (Prince et al., 2016).

The etiology of dementia remains unclear. Its increasing prevalence rate contributes to both a health and social problem, resulting in heavy caregiver burdens and economic impacts to the societies (2012 Alzheimer’s Disease Facts and Figures, 2012). Age and female gender are two major risk factors for Alzheimer’s disease (AD), which is the main cause of dementia; two-thirds of elderly people with AD are women. Even regarding the difference in longevity, studies suggest that women are still at a higher risk (Prince et al., 2016). Precision medicine approaches have advanced our understanding of the development and treatment of AD dementia. However, gender has not yet been adequately addressed by many of these approaches. More attention to gender differences will improve outcomes for people with dementia (Nebel et al., 2018). Previous research on NMDAR function has focused on cognition, particularly learning and memory. The current article focuses on mood or other psychological symptoms rather than memory.

## Behavioral and Psychological Symptoms of Dementia is Common and Detrimental in Demented Patients

One of the most troublesome domains of treating dementia is the behavioral and psychological symptoms of dementia (BPSD). The term “BPSD” was first described in late 1980s, and was then defined as “a term used to describe a heterogeneous range of psychological reactions, psychiatric symptoms, and behaviors occurring in people with dementia of any etiology” in the 1996 International Psychogeriatric Association (IPA) consensus conference (Finkel, 2003). BPSD can be classified into four domains: (1) disorders of thought content, including delusions, suspiciousness, etc. (Burns et al., 1990a); (2) disorders of perception, including misidentification syndromes, hallucinations, etc. (Burns et al., 1990b); (3) disorders of mood, including elevated mood, anxiety, depression, etc. (Burns et al., 1990c); and (4) disorders of behavior, including agitation, aggression, wandering, binge-eating, hyperorality, sexual disinhibition, urinary incontinence, etc. (Burns et al., 1990d).

BPSD is common among patients with dementia. Sixty-four percent of dementia patients revealed BPSD at initial evaluation (Devanand et al., 1997) and 90% of them had BPSD over the whole dementia course (Steinberg et al., 2008). In Taiwan, the prevalence rate of all types of BPSD in patients with AD is around 20–60%, varying with different symptoms (Fuh, 2006). About 30–60% patients with AD have delusion, 21–26% hallucination, 35–76% anxiety, 22–50% depression, and 26–61% sleep abnormalities (Fuh, 2006).

The manifestations of BPSD vary with different types and stages of dementia. Mood symptoms are usually more common while psychotic symptoms are less common in vascular dementia (VaD) (O’Brien, 2003). In Eastern Asia, patients with AD had a high incidence of anxiety/phobia (61.2%) and people with VaD had more paranoid and delusional ideation (71.9%) and affective disturbance (46.9%) (Chiu et al., 2006). Depression is more commonly observed in early stages of dementia while psychosis occurs more often in later stages (Paulsen et al., 2000; Savva et al., 2009). Furthermore, the severity of BPSD is often associated with the stage of dementia (Thompson et al., 2010). It is believed that BPSD without adequate treatment leads to poorer prognosis of dementia (Bourgeois et al., 1996; Shah and Allen, 1999; Brodaty et al., 2003; Huang et al., 2012).

## No Current Medication is Approved by Food and Drug Administration for Treating Behavioral and Psychological Symptoms of Dementia

Despite the high prevalence rate of BPSD and its hazards in patients with dementia, there has not yet been medication that is effective and approved for the treatment of BPSD by the Food and Drug Administration (FDA) (Sink et al., 2005). The efficacy of antipsychotics for the treatment of BPSD is scanty (Schneider et al., 2006). In addition, there is safety concern for antipsychotics use in BPSD (Lee et al., 2004; Schneider et al., 2005). Furthermore, atypical antipsychotics may worsen the cognitive decline in patients with AD (Vigen et al., 2011), implying that antipsychotics may not be the best remedy for BPSD.

The efficacy of acetylcholinesterase inhibitors (AChEIs), which are the current main medication for AD, for treating BPSD is controversial (Huang et al., 2012).

## N-Methyl-D-Aspartate Receptor and the Pathogenesis of Dementia and Behavioral and Psychological Symptoms of Dementia

N-methyl-D-aspartate receptors (NMDARs) exert multiple activities including two opposite ones: neurotoxicity and neurotrophic effects. Both NMDAR hypofunction and excitotoxicity are implicated in neurodegeneration. NMDAR activation is critical for synaptic plasticity, learning, and memory (Takehara et al., 2004; Zhao et al., 2005; Nakazawa et al., 2006; Gardoni et al., 2009). Attenuation of NMDAR neurotransmission can result in loss of neuronal plasticity and cognitive deficits (Collingridge and Bliss, 1995; Hawasli et al., 2007). Moreover, hypo-NMDAR function induced by NMDAR antagonists is neurotoxic, accounting for deterioration and brain atrophy (Olney and Farber, 1995).

NMDAR plays a vital role in the pathogenesis of AD. Compared with healthy controls, individuals with AD have fewer NMDARs in the frontal cortex and hippocampus (Procter et al., 1989), lower CSF concentrations of excitatory amino acids (Martinez et al., 1993), lower serum levels of D-serine (Hashimoto et al., 2004), and reduced D-aspartate uptake (Lowe et al., 1990). In a mouse model of AD, expression of surface NMDARs decreases in neurons (Snyder et al., 2005). Amyloid-β peptide (Aβ), which is the pathological hallmark for AD, can impair NMDAR signal transduction and synaptic function (Shankar et al., 2007; Yamin, 2009; Cisse et al., 2011). Apolipoprotein E4, an amyloid binding protein isoform related to the AD risk, also decreases NMDAR functions in patients with AD (Chen et al., 2010). Loss of presenilins reduces NMDAR-mediated responses and synaptic levels of NMDAR subunits, thereby affecting both short- and long-term plasticity in AD pathogenesis (Pimplikar et al., 2010).

## N-Methyl-D-Aspartate Receptor Inhibiting Agents

Memantine, an NMDAR partial antagonist and a drug for treating moderate-to-severe AD, had conflicting data in the treatment of neuropsychiatric symptoms in dementia from randomized controlled trials (Reisberg et al., 2003; Tariot et al., 2004; Sink et al., 2005). In subsequent individual studies and pooled analyses (Gauthier et al., 2005, 2008; Wilcock et al., 2008), memantine had some benefits in the treatment of irritability/liability, agitation/aggression, and psychosis in patients with AD, but stronger evidence from randomized controlled trials for BPSD is still lacking (Ballard et al., 2009). Lately, memantine’s effect for BPSD has been found to be boosted by combination of citalopram, an antidepressant (Zhou et al., 2019). Of note, an initial reason for the use of memantine to treat AD is the hypothesis that activity of the NMDAR could be a mechanism for cell death (Foster et al., 2017). Thus, a potential fear is that enhancing NMDAR function would adversely affect the trajectory of dementia. This may be true for a subclass of dementia. On the other hand, another hypothesis (Foster et al., 2017) also indicates that NMDAR hypofunction is more detrimental to the progression of AD and that the use of memantine as a treatment may be more detrimental, producing cognitive impairments.

Inhibition of NMDAR function by NMDAR antagonists, such as ketamine or phencyclidine, produces psychotic/behavioral symptoms or relevant physiological reactions (Oranje et al., 2002; Carlen et al., 2012; Lin et al., 2012). However, the NMDAR antagonist ketamine has been shown to exhibit antidepressant effects (Duman, 2018). Since depression is one of the BPSD symptoms, whether ketamine and its derivatives, as rapid-acting antidepressants, are beneficial for BPSD treatment deserves further studies (Steenblock, 2018).

NMDAR dysfunction is also involved in ischemic stroke and vascular dementia. Although some studies revealed serum glutamate elevation in acute phase of ischemia stroke (Gusev et al., 2000; Marcoli et al., 2004), this increment of glutamate was only found 10–30 min after ischemic injury (Benveniste et al., 1984). Similarly, NMDAR hyperfunction occurs very briefly after brain injury; soon after the acute glutamate elevation, profound NMDAR hypofunction ensues and lasts for >7 days (Biegon et al., 2004). In fact, trials using NMDAR antagonists in the treatment of stroke have failed (Ikonomidou and Turski, 2002).

## N-Methyl-D-Aspartate Receptor Enhancing Agents

Enhancing NMDA neurotransmission can improve memory and behavior symptoms of both dementia and schizophrenia (Goussakov et al., 2010; Lane et al., 2013). Clinical characteristics of BPSD, such as hallucinations, delusions, disorganized speech, and disturbing behavior, resemble positive symptoms of schizophrenia. Social withdrawal, apathy, alogia, and avolition, which resemble negative symptoms in schizophrenia, and behavior, sleep, or affective problems, are also frequently seen in patients with schizophrenia.

Augmentation through the NMDA-glycine site, a co-agonist site, is preferred to avoid the excitotoxicity (Coyle and Puttfarcken, 1993; Collingridge et al., 2013; Hackos and Hanson, 2017; Yao and Zhou, 2017; Hsu et al., 2018). Clinically, D-cycloserine, a partial agonist of the NMDA-glycine site, can improve cognitive functions of demented patients (Schwartz et al., 1996; Tsai et al., 1999). There are also several studies that suggest no benefit of D-cycloserine in AD patients (Randolph et al., 1994; Fakouhi et al., 1995; Tsai et al., 1998). Several possible reasons may explain the discrepancies; the lack of effect may be due to the dose or symptoms examined. Alternatively, the effects of D-cycloserine may depend on the stage of dementia. Since D-cycloserine appears to have benefits in improving cognition (Kalisch et al., 2009; Onur et al., 2010; Kuriyama et al., 2011a,b; Feld et al., 2013), it may have differential effects on mood and learning depending on the stage of dementia.

Since D-serine is more potent than D-cycloserine and glycine as the glycine co-agonist site of the NMDAR (Heresco-Levy, 2005; Lin et al., 2012), one method to enhance NMDAR function is to inhibit activity of D-amino acid oxidase (DAAO), which is responsible for degrading D-serine and D-alanine (Fukui and Miyake, 1992; Vanoni et al., 1997). One of the candidates of DAAO inhibitors is benzoic acid and its salt, sodium benzoate. They can inhibit DAAO activity and thereby raise synaptic concentrations of D-serine both *in vitro* and in animal studies (Van den Berghe-Snorek and Stankovich, 1985).

Several clinical trials have shown the potential of NMDAR-enhancing agents [for example, sarcosine (a glycine transporter I inhibitor) and sodium benzoate] in alleviating psychotic symptoms of schizophrenia (Lane et al., 2005, 2006, 2008, 2010, 2013; Lin et al., 2018b), in treating major depressive disorder (Huang et al., 2013), in decreasing oppositional defiant disorder symptoms of attention deficit hyperactivity disorder (Tzang et al., 2016), and in reducing neuropsychiatric symptoms of Parkinson’s disease with dementia (Tsai et al., 2014).

In a 6-week, randomized, double-blind, placebo-controlled trial in patients with schizophrenia (<65 year old), sodium benzoate (1 g/day) adjunctive therapy was significantly better than placebo in reducing positive and negative symptoms and in improving Global Assessment of Functioning, and revealed favorable safety (Lane et al., 2013). The effect size of sodium benzoate treatment for Positive and Negative Syndrome Rating Scale (PANSS) total score from baseline to endpoint was 1.26, much higher than effect size (0.51) of sarcosine adjuvant therapy for the PANSS total score in chronic schizophrenia patients (Tsai et al., 2004). It is noteworthy that sodium benzoate treatment was significantly better than placebo in improving cognitive functions, such as processing speed and visual memory (Lane et al., 2013). In another clinical trial on mild cognitive impairment or mild AD, a total of 60 patients were randomized into sodium benzoate or placebo group. The patients also tolerated sodium benzoate 250–1,500 mg/day well without evident side effects. Interestingly, the patients taking sodium benzoate improved more in Alzheimer’s Disease Assessment Scale-cognitive subscale (ADAS-cog) and other cognitive assessments than placebo (Lin et al., 2014).

Of note, a single nucleotide polymorphism (rs2153674) in the G72 (D-amino acid oxidase activator, DAOA, responsible for metabolism of D-serine) gene is associated with the occurrence of psychotic symptoms in patients with AD (Di Maria et al., 2009). In addition, affinity of the glycine recognition sites of NMDARs was related with the anxiety tone, one domain of BPSD, in patients with AD (Tsang et al., 2008). Therefore, it is possible that NMDAR-enhancing agents, which have been demonstrated to be effective in treating schizophrenia, depression, and other psychiatric symptoms, could also be used in the treatment of BPSD.

Moreover, stimulation of NMDARs 24 and 48 h after brain injury could attenuate neurological deficits and improve cognitive performance, implying that NMDAR function is crucial for neural repair in subacute or chronic stroke (Biegon et al., 2004). The aforementioned studies suggest the potential use of DAAO inhibitors for the treatment of BPSD.

## Gender Difference in N-Methyl-D-Aspartate Receptor Function

Age and female gender are two major risk factors for AD; two-thirds of elderly people with AD are women. Even regarding the difference in longevity, studies suggest that women are still at a higher risk (Prince et al., 2016). However, gender has not yet been adequately addressed by many of these approaches. More attention to gender differences will improve outcomes for demented people (Nebel et al., 2018).

A previous study showed that female rats were much more susceptible to NMDAR modulation than males (Honack and Loscher, 1993). Another study found that the average density of NMDAR currents was 2.8-fold larger in dorsal root ganglia of female rats than that of male rats, and that addition of 17-ß-estradiol (E2) increased NMDAR currents by 55% in female neurons, but only 19% in male, indicating sex differences in the activity and estrogen modulation of NMDAR (McRoberts et al., 2007). Further, estrogen also plays a role in NMDAR function during aging (Vedder et al., 2014; Bean et al., 2015). E2 treatment can enhance the long-term potentiation (LTP) magnitude at CA3-CA1 synapses, NMDAR/AMPAR ratio, GluN2B-mediated NMDAR current, hippocampal CA1 dendritic spine density, and novel object recognition (NOR), a task that requires hippocampal NMDARs, in female rats during a critical period between 9 and 15 months, but not at 19 months post-ovariectomy (OVX) (Smith et al., 2010; Vedder et al., 2014).

Sex hormones were found to modulate hippocampal NMDAR expression in mice (McCarthny et al., 2018), and interact with circulating antioxidants in human blood (Bellanti et al., 2013). Noteworthily, benzoic acid ester of estrone, a precursor of estradiol, has prolonged duration of action (Labhart, 2012), suggesting that benzoate may interact with female sex hormone (Lemini et al., 1997).

Whether benzoate can improve cognitive function and the behavioral symptoms in patients with BPSD in a gender-specific manner deserves investigation. Further study is needed to verify the possible mediating roles of sex hormones in benzoate effect for dementia and its associated BPSD.

## Hunting for Peripheral Biological Markers of Dementia

At present, the diagnosis of dementia mainly relies on clinical manifestation. There was no satisfactory laboratory test from the peripheral approach for the diagnosis of dementia. There have been lots of postmortem brain studies in fields of AD and related neurodegenerative disorders (Chen et al., 2001; Stewart et al., 2001). It was highly concerning that RNA expressions might be affected by many factors (e.g., coma. hypoxia) under postmortem condition (Tomita et al., 2004). A peripheral measurable marker is needed, to enable a simple, more rapid, and more accurate diagnosis and monitoring (Ilani et al., 2001; Lin et al., 2017a).

It is proposed that white blood cells and lymphocytes may serve as neural probes since there are similarities of signal transduction and receptor expression between peripheral blood cells and neurons/glia (Gladkevich et al., 2004). A blood-derived sample will be a more feasible alternative to brain tissue biopsy if the gene expressions are synchronized in both (Tsuang et al., 2005). For example, Hye et al. reported that glycogen synthase kinase-3 was increased in both AD and mild cognitive impairment patients (Hye et al., 2005). However, some molecules would be not suitable for serving as biomarkers, due to the large overlap between patient and control groups.

## Potential Peripheral Predictors for Treatment Response of N-Methyl-D-Aspartate Receptor Enhancers

Precision medicine approaches have advanced our understanding of the development and treatment of dementia. Many genes which lie in different pathways were found to be associated with susceptibility of dementia and/or psychosis. Genes on the pathways, which are associated with the metabolism of D-amino acids, glycine and glutamate, may be able to regulate the NMDAR function.

### D-Amino Acids Metabolism

D-amino acid oxidase activator (DAOA, or named G72) protein regulates DAAO activity (Goldberg et al., 2006), enhances metabolism of D-serine and D-alanine, and can attenuate NMDAR neurotransmission. D-serine is generated from L-serine by serine racemase (SRR) (Wolosker et al., 1999) and degraded by DAAO (Nagata, 1992). Over the past few years, more than 30 studies have demonstrated the association of DAAO and G72 with schizophrenia (Boks et al., 2007). Diminished D-serine along with elevation in L-serine also suggests the dysfunction of SRR activity (Hashimoto et al., 2003). DAAO is implicated in oxidative stress (Stegman et al., 1998; Lu et al., 2012). Studies indicated that the DAAO level in peripheral blood increased with the severity of cognitive deficits in the elderly (Lin et al., 2017b) and decreased after 6-week treatment of sodium benzoate (a DAAO inhibitor) in patients with schizophrenia (Lin et al., 2018a). It is hypothesized that G72, DAAO, and SRR, which regulate the metabolism of the main co-agonist of the NMDAR, D-serine, are associated with dementia and its BPSD.

### Glycine Metabolism

Glycine, a co-agonist of the NMDAR, is abundant throughout the brain and serves as a major inhibitory neurotransmitter in the hindbrain. Serine hydroxymethyltransferase (SHMT) is the enzyme which cleaves serine into glycine (Cossins et al., 1976). The activity of SHMT was significantly lower in psychotic individuals than in nonpsychotic ones (Waziri et al., 1985). Phosphoserine aminotransferase (PSAT) enzyme accounts for the serine biosynthesis (Pestka and Delwiche, 1981). Patients with PSAT deficiency manifest a broad spectrum of neuropsychiatric symptoms clinically (Hart et al., 2007). Glycine C-acetyltransferase, also known as GCAT, acts in concert with L-threonine 3-dehydrogenase (TDH) in the degradation of threonine to form glycine (McGilvray and Morris, 1969). Aminomethyltransferase (AMT) is an enzyme that catabolizes the creation of methylenetetrahydrofolate. It is part of the glycine decarboxylase complex.

### Glutamate Metabolism

Glutamate is the most abundant amino acid neurotransmitter in the mammalian brain. Glutamatergic neurotransmission has drawn attention for its role in the pathophysiology of many mental illnesses (Lin et al., 2012). The extracellular concentration of glutamate is regulated by the action of transporter proteins, which include glial high-affinity glutamate transporter, member 3 (SLC1A3) (Kanai and Hediger, 2004) and neutral amino acid transporter (ASCT1) (Weiss et al., 2005). Glutamate receptor, metabotropic 3 (GRM3) (Carter, 2007) and glutamate receptor, ionotropic, kainate 1 (GluR5) (Wisden and Seeburg, 1993) are among the genes related to the glutamatergic neurotransmission systems. Glutamate decarboxylase 1 (GAD1), encoding the 67-kDa isoform of glutamate decarboxylase, is the key enzyme for GABA biosynthesis and is expressed at altered levels in postmortem brain of subjects diagnosed with schizophrenia and related psychotic disorders (Straub et al., 2007).

Increased oxidative stress also contributes to aging processes and neurodegenerative diseases (Gallagher et al., 1996; Serrano and Klann, 2004; Butterfield and Halliwell, 2019), while free radicals damage cells and tissues (Harman, 1956). Antioxidants may help to prevent and reverse cognitive deficits induced by free radicals (Guerrero et al., 1999; Bickford et al., 2000; Tardiolo et al., 2018). Studies indicate a link among age-related NMDAR dysfunction, oxidative stress, and senescence and related cognitive decline (Guidi et al., 2015; Kumar, 2015).

## Summary

BPSD appears the hardest-to-treat domain of dementia. Non-pharmacological approaches are the mainstream treatment; however, psychotropics are still needed for a substantial portion of patients. While second-generation antipsychotics have been widely used for the treatment for BPSD, their adverse effects generally offset the benefits. To date, no pharmacological approach has been approved for the treatment of BPSD.

Lately, NMDAR activating strategies, such as DAAO inhibition, have been demonstrated to benefit early-phase dementia as well as psychotic disorders such as schizophrenia. Whether such a novel medical route can also improve BPSD (or a fraction of it, with the aid of molecular precision medicine) deserves studies.

Since BPSD is difficult to treat, it is important to identify subpopulations that tend to respond to certain treatments. Recent studies suggest that the NMDAR expression may be different between female and male species. Genes involved in the pathways associated with the regulation of NMDAR might be altered in BPSD, and have potential to be developed as biomarkers for detecting dementia and predicting the treatment response.

In summary, the review addressed the NMDAR-related modulation and precision medicine in BPSD. Future studies examining the NMDAR modulators with the aid of potential biomarkers to tailor the treatment for individualized patients with BPSD will advance the treatment of BPSD.

## Author Contributions

C-HL and H-YL determined the outline, reviewed the literature, and wrote and approved the manuscript.

### Conflict of Interest Statement

The authors declare that the research was conducted in the absence of any commercial or financial relationships that could be construed as a potential conflict of interest.
